# Laparoscopic Roux-En-Y gastric bypass versus one anastomosis (loop) gastric bypass for obesity: A prospective comparative study of weight loss and complications

**DOI:** 10.1016/j.amsu.2020.04.040

**Published:** 2020-05-18

**Authors:** Abdalla Mustafa, Nayer N.H. Rizkallah, Nehemiah Samuel, Shlokarth Balupuri

**Affiliations:** a Sunderland Bariatric Unit, Sunderland Royal Hospital, Sunderland, United Kingdom; b General Surgery Department, Cairo University, Egypt

**Keywords:** Laparoscopic Roux en y (LRYGB), One anastomosis gastric bypass (OAGB), Excess body weight, Total weight loss

## Abstract

**Introduction:**

Most Bariatric units perform Laparoscopic Roux-en-Y Gastric Bypass (LRYGB) and One Anastomosis Gastric Bypass (OAGB) for weight loss and metabolic purposes with satisfactory results and low complication profile.

**Objectives:**

This study compares LRYGB and OAGB outcomes in a high volume single bariatric unit.

**Methods:**

Data was collected prospectively and analysed retrospectively for all LRYGB and OAGB performed between Jan 2014 to Dec 2016. The follow up period was for 2 years. Patients who were lost to follow up or had prior bariatric procedure were excluded. Excess weight loss percentage (EWL %), total weight loss percentage (TWL %) and post-operative complications were compared in both groups.

**Results:**

1268 procedures performed. 113 patients were excluded. At 2 years, for LRYGB and OAGB groups mean TWL % was 31% and 35.4% respectively (P < 0.0001); and mean EWL % was 70.1% and 74.8% respectively (P = 0.0119). Gastroesophageal reflux symptoms were higher in OAGB group 17 (8.5%), with 7 patients needing further surgery, versus 26 (2.7%) in LRYGB (P = 0.0003). There was no difference in incidence of marginal ulcers between LRYGB and OAGB 2.7% vs 5% respectively (P = 0.1115). Internal hernia was seen only in LRYGB patients, 22 (2.2%). There was no significant difference in the re-operation rates following LRYGB 52 (5.4%) and OAGB 16 (8%) (P = 0.1824).

**Conclusion:**

OAGB had superior short-term weight loss and low complications profile. Both procedures demonstrated no difference in either marginal ulcers or re-operation rates. Reflux symptoms have remained a major side effect of OAGB.

## Highlights

• One anastomosis gastric bypass has shown to have a safe operative profile and effective weight loss outcomes.
• No difference in marginal ulcerations or reoperation rate between One anastomosis gastric bypass and Laparoscopic Roux en y.
• We recommend strict selection criteria for patient undergoing OAGB.

## 1 Introduction

The problem of obesity is one of epidemic proportions and a cause of significant health concern in both developed and developing countries [1,2]. Morbid obesity (MO) affects multiple systems in the body and its subsequent complications leads to an overall decrease in life expectancy. Surgery has clearly demonstrated to provide sustainable weight loss and health improvement over the long term [3]. This has hence led to development of surgical weight management strategies. The surgical weight loss mechanism is generally considered to target diet restriction, malabsorption, or a combination of these mechanisms. Several standard surgical procedures such as Laparoscopic adjustable gastric band (LAGB), vertical sleeve gastrectomy (LSG), Roux-en-Y- gastric bypass (LRYGB) and biliopancreatic diversion with (duodenal switch) are being performed with varying degrees of success and complications.

Over the years, Laparoscopic Roux en Y Gastric Bypass (LRYGB) is a commonly performed bariatric surgery which has stood the test of time by achieving good long-term results with weight loss and comorbidities resolution [4]. However, while some studies have shown relatively higher complication rates both surgical and malabsorptive with LRYGB when compared to other commonly performed bariatric procedures [[5], [6], [7]]; other studies have shown no difference at all in the short or long term [8,9]. As a primary bariatric procedure, One Anastomosis Gastric Bypass (OAGB) (first described by Rutledge) has the advantage of shorter operation time and lower post-operative complication rates [8,10]. It has rapidly become an established standard procedure for weight loss in obese patients [11]. The aim of this study was to compare weight loss outcomes and complications of both LRYGB and OAGB standard procedures in a high-volume bariatric unit in the UK.

## 2 Methods

This study is a comparative study of all primary Laparoscopic Roux-En-Y Gastric Bypass (LRYGB) and One Anastomosis Gastric Bypass (OAGB) procedures performed between January 2014 and December 2016 in a single bariatric unit. The data was analysed retrospectively from a prospectively maintained electronic database. The follow up period was 2 years. Exclusion criteria were prior bariatric procedure and any revisional surgery. Those who failed to complete the 2 year follow up period were excluded from further analysis.

Protocol based bariatric assessment including dietetic assessment and psychological evaluation were used in the pre-operative period. Bariatric Multi-disciplinary meeting review was mandatory for all patients prior to surgery. Pre-operative Oesophago-gastroduodenoscopy (OGD) was routinely performed for all patients prior to surgical intervention as per protocol to evaluate the presence of hiatus hernia, oesophagitis and exclude peptic ulcer disease. Patient's Smoking status was also confirmed before surgery and procedures were offered to those who stopped smoking.

Weight and Body Mass Index (BMI) were recorded at the initial clinical assessment and subsequent follow-up visits. The 2-year weight loss result was calculated by Excess Weight Loss percentage (EWL %) and Total Weight Loss percentage (TWL %). Any post-operative complications were recorded during the entire period of follow up. The resources used to verify events were entries in patient's electronic notes, Clinic letters and Radiological Imaging. Emphasis was laid on evaluation of post-operative marginal ulcers and imaging for re-admitted patients with suspected internal hernia or obstruction. Furthermore, all re-operations notes were reviewed to gather intra-operative findings in the post LRYGB and OAGB patients. All revisional surgeries as well as reversal of bypass were recorded, including their clinical indications. This study is being reported in line with the strengthening the reporting of cohort studies in surgery (STROCSS) criteria [12].

### 2.1 Bariatric procedures

In our unit, LRYGB and OAGB are performed by 6 bariatric surgeons. In both procedures, the surgeon would be standing between the patient's legs and one assistant is on the left-hand side of the patient. Five ports are used (3 × 12 mm ports and 2 × 5 mm ports). Nathanson retractor is routinely used to retract the left lobe of the liver.

#### 2.1.1 LRYGB

The dissection starts at the angle of His. Retro-gastric tunnel is fashioned at the level of the second left gastric artery using Ethicon Harmonic scalpel. An Endo GIA tristapler device with 45 mm cartridge is fired across the stomach followed by three 45 mm cartridges fired vertically towards the dissected angle of His. This step ends by forming a small gastric pouch. A gastrotomy is formed at the most dependant part of the pouch using harmonic scalpel. The omentum is reflected to expose the DJ flexure. A 50 cm biliary limb is anastomosed, in an antecolic position, to the pouch using 45 mm tristapler cartridge while the gastroenterotomy is closed with Ethicon 2/0 vicryl in one or two continuous layers. A 150 cm alimentary limb is measured and jejuno-jejunal anastomsis is performed with 45 mm tristapler cartridge. The enterotomy is closed by 2/0 vicryl in one or two continuous layers. An Endohernia stapler device is then used to close the Peterson's space and the meso-mesenteric space. The omega loop is then divided using laparoscopic staplers. Methylene blue leak test is performed to check the gastro-jejunal anastomosis to confirm patency and exclude leakage.

#### 2.1.2 OAGB

The steps are similar to LRYGB with some differences. The retro-gastric tunnel is fashioned at the level of the incisura. The stomach is cross stapled with 45 mm tristapler cartridge and then the gastric pouch is fashioned vertically till the angel of His using 5 or 6 tristapler cartridges (Longer pouch). The biliary limb is 150 cm long and the ante-colic gastro-jejunal anastomosis is created by a laparoscopic stapler. Gastro-enterotomy is closed using 2/0 vicryl in one or two continuous layers. Peterson's space is closed by an Endohernia stapler device. Methylene blue leak test is also performed.

## 3 Statistical analysis

Data were recorded onto a dedicated database (Microsoft® Excel; Microsoft, Redmond, Washington, USA). Continuous data were first tested for normality using histograms. Based on distribution testing results, the continuous variables were presented as either mean with standard deviation (SD) or median with inter quartile range (IQR) and analysed using the independent sample *t*-test or Kruskal Wallis ANOVA test for unrelated samples respectively. Categorical data were analysed using the Chi-Square test. Statistical analysis was done using SPSS® version 21.0 (SPSS, Chicago, Illinois, USA). A p-value less than 0.05 was considered to be significant.

## 4 Results

### 4.1 Demographics

A total of 1268 patients were identified who underwent LRYGB or OAGB during the study period and met the inclusion criteria. 113 patients (8.9%) were lost to the 2 year follow up and therefore excluded from further analysis.

Of the 1115 (957 LRYGB, 198 OAGB), 968 (83.8%) were females and 187 (16.2%) were males. The mean age at surgery was 45.3 (18–72) for LRYGB group and 44.4 (16–78) years for OAGB group. All procedures were performed laparoscopically. At initial clinical assessment, the mean (SD) weight and BMI recorded for LRYGB were 128.7 (23.3) kg and 46.4 (6.9) kg/m2 respectively while those for OAGB were 139.4 (30.8) kg and 48.8(7.8) kg/m^2^ respectively (Table 1).

**Table 1:** Demographics, initial weight & BMI.

Patient Characteristics	LRYGB *(n* = 957)	OAGB *(n* = 198)	P value
Females	87.3% (*n* = 836/957)	66.6% (*n* = 132/198)	<0.001
Age at the time of Surgery Mean	46.2 SD 11.1	44.5 SD 11.8	0.056
Initial Weight Mean	128.7 kg SD 23.3	139.4 kg SD 30.8	<0.001
Initial BMI Mean	46.4 kg/m^2^ SD 6.9	48.8 kg/m^2^ SD 7.8	<0.001

### 4.2 Weight loss at 2 years

Over the 2 year follow up period a mean (SD) weight loss of 41.2 (15.8) Kg in the LRYGB group and 49.3 (19.2) Kg in OAGB group (P < 0.001) was seen.

**Fig. 1:**
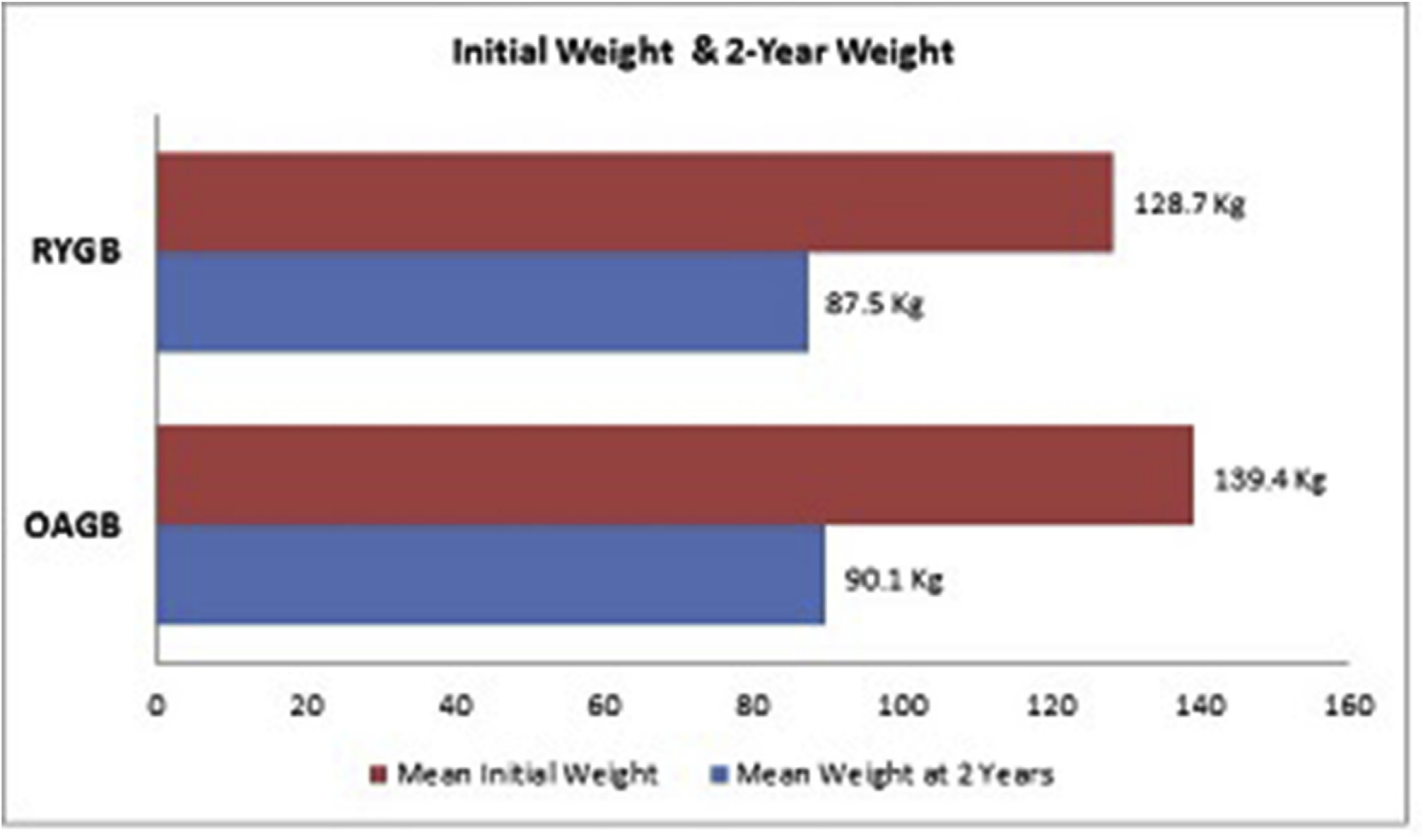
Initial weight and 2-year weight.

**Fig. 2:**
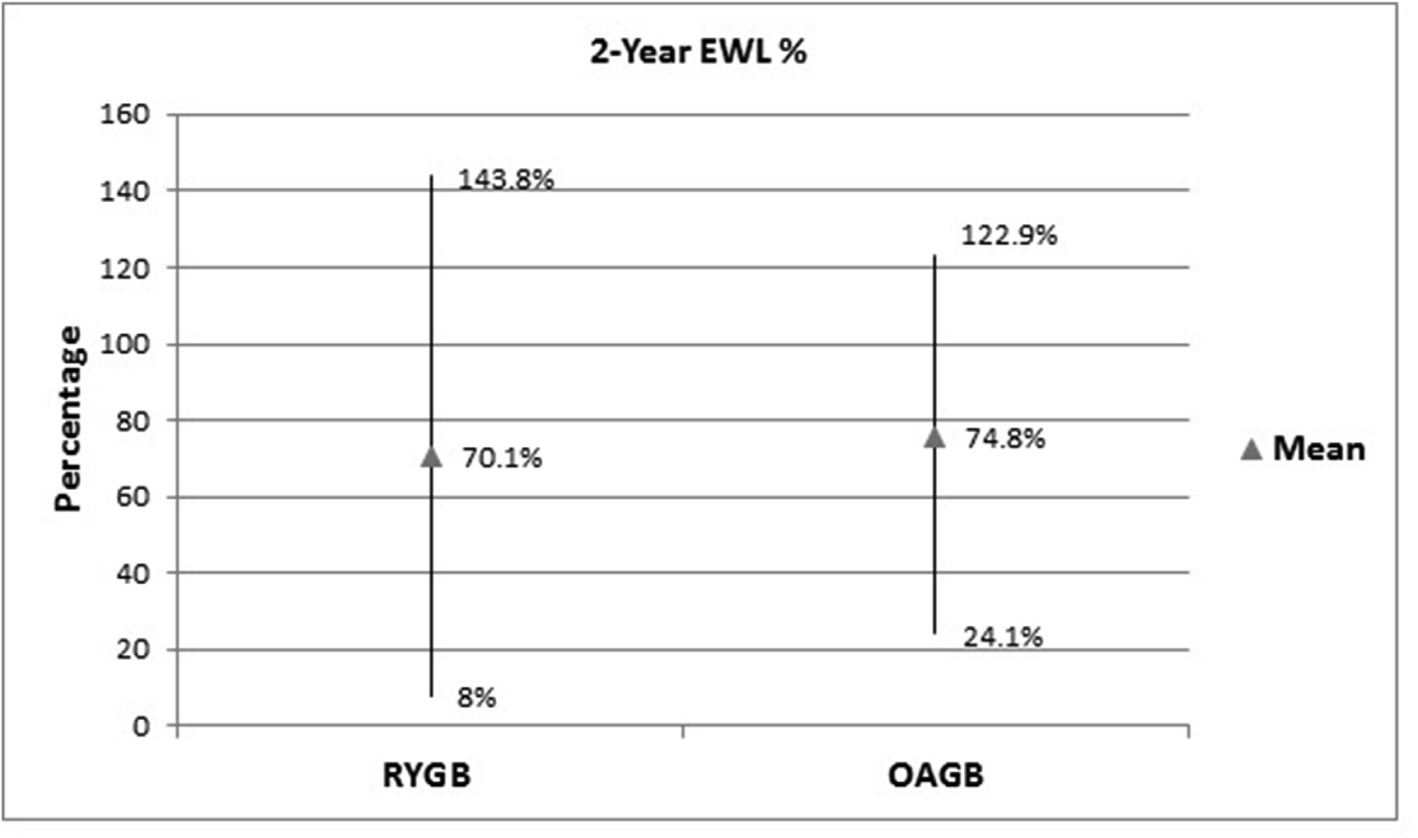
2-year EWL%.

The calculated mean TWL% and mean EWL% were 31.1% (9.7%) and 70.1% (23.2%) respectively for LRYGB patients while for OAGB patients were 35.1% (9%) and 74.5% (19.3%) respectively (Table 2), (Fig. 1, Fig. 2).

**Table 2:** Weight loss data at 2 years.

Weight Loss	LRYGB	OAGB	P value
Mean Weight loss in kg	41.2 kg SD 15.8	49.3 kg SD 19.2	<0.001
Mean Excess Weight Loss	70.1% SD 23.2	74.5% SD 19.3	0.011
Mean Total Weight Loss	31.1% SD 9.7	35.1% SD 9	<0.001

### 4.3 Gastroesophageal reflux

Post-operatively reflux symptoms were reported in 2.7% (26/957) of LRYGB group and 8.5% (17/198) in OAGB group. The incidence of reflux symptoms in the OAGB group was significantly higher (P < 0.001). Subset analysis showed only one (0.1%) of the twenty-six patients in LRYGB group requiring further surgery to repair hiatus hernia. However, in OAGB six (3%) patients had revisional surgery to LRYGB for severe symptoms control and one (0.5%) patient underwent hiatus hernia repair. Rest of the ten (5%) patients’ symptoms were treated medically.

### 4.4 Marginal ulcers

2.7% (26/957) LRYGB patients developed marginal ulceration, of which only one (0.1%) had to have reversal procedure for symptomatic intractable marginal ulcer. In the OAGB group, 5% (10/198) developed marginal ulceration. Two (1%) patients required conversion to LRYGB for perforation and anastomotic stricture. The difference in marginal ulcers occurrence between the two groups was not found to be statistically significant (p = 0.111).

### 4.5 Re-operations

#### 4.5.1 LRYGB

Internal hernia cases were only identified in LRYGB patients. A total of 2.2% (22/957) patients had re-operations for internal hernia repair. Jejunal mesenteric defect was found in 1.5% (15/957) patients and Peterson's defect in 0.6% (6/957) patients. In one patient (0.1%), both spaces contributed to the incidence of internal hernia.

On diagnostic laparoscopy for unexplained abdominal pain, 15(1.5%) patients were found to have open internal hernia spaces, with no bowel herniation at the time, (Nine Jejunal mesenteric space, one Peterson's space and five both spaces), three patients had adhesiolysis while one patient had hiatus hernia which was then repaired.

Small bowel obstruction was recorded in 1(0.1%) patient secondary to adhesions requiring adhesiolysis and 1(0.1%) patient required revision of Jejuno-Jejunal anastomosis to relieve the obstruction.

Iatrogenic Bowel Injury was reported in one patient who required early return to theatre for laparoscopic repair. Two patients underwent laparoscopic washout of intra-abdominal collection few months after initial surgery. Postoperative bleeding was encountered in only two patients. One was controlled after re-laparoscopy and washout while the second one was an intra-luminal bleed which was controlled endoscopically by an OGD performed under General anaesthesia.

Jejuno-Jejunal anastomotic complications were seen in 3 (0.3%) patients, One had revision of Jejuno-Jejunal anastomosis following perforation while two other patients had it revised for obvious narrowing at the site of anastomosis. Reversal of LRYGB was performed on 1(0.1%) patient for intractable marginal ulcer (Table 3) as mentioned above.

**Table 3:** Re-operations.

Causes	LRYGB *n* 957	OAGB *n* 198	P Value
Internal Hernia	22 (2.2%)	0 (0%)	0.022
Diagnostic Laparoscopy	19 (1.9%)	4 (2%)	>0.05
Small Bowel Obstruction	2 (0.2%)	2 (1%)	0.138
Reversal	1 (0.1%)	0 (0%)	>0.05
Iatrogenic injury	1 (0.1%)	1 (0.5%)	>0.05
Intra-abdominal collection	2 (0.2%)	1 (0.5%)	0.4315
Bleeding	2 (0.2%)	0 (0%)	>0.05
Other Causes	JJ Complications:3 (0.3%)	Conversion to RYGB:8(4%)	
Total	52 (5.4%)	16(8%)	0.1824

#### 4.5.2 OAGB

Diagnostic laparoscopy for unexplained abdominal pain was performed in 4 (2%) patients, two had adhesiolysis and one patient also had hiatus hernia repair, while two patients had no identifiable intra-abdominal pathology. Small bowel obstruction was recorded in 2 (1%) patients secondary to band adhesions requiring adhesiolysis. Laparoscopic washout of intra-abdominal collection was required in one patient.

Gastric obstruction was reported in one patient. This required early return to theatre to relieve antral stenosis of the gastric remnant.

Conversion to LRYGB was performed on 8 (4%) patients. Six, due to severe gastro oesophageal reflux symptoms which did not respond to medical treatment. Of the two patients troubled with marginal ulcers, one had revision to RYGB on an emergency basis following perforation while the second patient was revised following stricture at the gastro-jejunostomy as a result of longstanding marginal ulcer (Table 3).

## 5 Discussion

This is a large observational cohort study of 1155 patients who underwent either of the two standard bariatric bypass procedures.

Only few limited studies have compared OAGB and LRYGB in terms of weight loss and complications profile. This was shown in a recent meta-analysis (Wang et al., 2018) which included only ten cohort studies and one randomised controlled trial with variable number of patients in each study [13]. Wang's study compared the effectiveness between the two procedures. It concluded that OAGB had a better weight reduction effect and recommended larger sample size studies to compare outcomes. In addition, OAGB has been shown to be effective alternative procedure of choice in super and morbid obese patients [14,15].

In our study, OAGB has shown superiority over LRYGB in weight loss in that the mean EWL% at 2 years was 74.8% for OAGB and 70.1% for LRYGB which was statistically significant. This is very much in keeping with results from other studies [13,16,17]. Another study by Disse et al. showed that EWL % was significantly greater in the OAGB group [18] where the initial BMI was matched and the weight loss data was collected at 6 months and 1 year postoperatively. A randomised controlled trial study by Lee WJ et al., in 2005, comparing both groups did show better weight loss results after OAGB compared to LRYGB at 2 years(16). The same author in a different study followed a cohort of 1657 patients who underwent both operations, over a follow up period between 1 and 10 years, and found at 5 years post-surgery the mean BMI was lower in OAGB than LRYGB (27.7 vs. 29.2, p < 0.05) and OAGB also had a higher excess weight loss than LRYGB (72.9 vs. 60.1%, p < 0.05) [7].

In our observational study, although the mean initial weight for OAGB was higher at 139.5 kg than the RYGB group at 128.7 kg, yet the OAGB group demonstrated better total and excess weight loss over the study period. In the past, an observed practice in our unit was to treat the higher morbidly obese and superobese patients with sleeve gastrectomy as part of a two-phase surgical weight loss management. However, over the years, more such patients were offered OAGB as a single intervention, which was also reflective of the surgeons experience beyond the learning curve when more patients underwent OAGB procedures. This might explain the observed significant finding of higher initial weight among the OAGB cohort.

Gastro-oesophageal reflux symptoms in the OAGB group were higher compared to LRYGB group in our study. Parmar et al., in a recent study has described similar incidence (2%) of patient reported reflux symptoms after OAGB(11). In a different study, OAGB patients who required further revisional surgery (20/32) were found to be due to symptomatic bile reflux [19]. In our study, seven patients failed to resolve with medical management and hence had to undergo further procedures. The relatively higher rates of post OAGB reflux has been observed despite all patients undergoing extensive medical & pharmacological history and routine gastroscopy to pre-operatively identify those who suffer from significant reflux due to hiatus hernia. Based on positive gastroscopy findings, patients were offered LRYGB and advised against OAGB. In this context, a possible explanation for the higher incidence could be due to new reflux symptoms in patients who had pre-operative asymptomatic small hiatus hernia and opted for OAGB after considering the benefits and risks of both bypass procedures. The effect of these should be further studied.

There was no statistical difference in rate of re-operations between the two groups (5.4% in LRYGB vs 8% in OAGB). Internal hernia was only observed in LRYGB. It is standard practice in our unit to routinely close the mesenteric and Petersen's hernial space with either mechanical fixation device or with sutures during LRYGB. The RCT comparing LRYGB and OAGB [16] with 40 patients in each arm showed superior results with OAGB. LRYGB had higher complications rates at 20% (eight patients) compared to 7.5% (three patients) seen with OAGB. This variation could be explained and observed in different bariatric unit with varied volume of workload and differences in surgeon's experience. In a large study of 2678 patients looking at post-operative complications following OAGB, findings were intraoperative and early complications rates of 0.5 and 3.1% respectively. Late complication rates of 10.1%, with follow-up of 62.6% at 5 years [20]. In our study, the relatively lower OAGB complication rates could be explained by the larger volume case load and the fact that all Bariatric surgeons undertake it routinely and are well past the learning curve.

Conversion from OAGB to LRYGB was performed in 8 (4%) patients, due to severe gastro-oesophageal reflux symptoms and marginal ulceration. In a recent study by Hussain et al., complications requiring revisional surgery after OAGB are uncommon (2.3%) [21]. Similar to our study, Johnson et al. showed indications for conversion of OAGB to LRYGB; out of 32 patients who required revisional surgery, 25 patients had bile reflux and intractable marginal ulcers. 21 patients underwent LRYGB and further 5 were planned to have revisional surgery in the future [19].

## 6 Conclusion

Both LRYGB and OAGB have shown good 2-year weight loss profiles with OAGB having a greater weight loss effect in comparison. Of the two techniques, there was no difference in the overall complication rates, marginal ulcerations and re-operations. Gastro-oesophageal reflux symptoms are relatively more common in OAGB patient with a proportion of patients requiring revisional surgery. On this basis, we recommend that strict selection criteria should be in place to exclude patients with hiatus hernia, gastro-oesophageal reflux symptoms or signs, prior to offering eligible patients OAGB procedure.

## Data statement

Data used in completing this work is confidential. If still required, data could be provided after removing the patient’s sensitive data.

## Funding

This research did not receive any specific grant from funding agencies in the public, commercial, or not-for-profit sectors.

## Provenance and peer review

Not commissioned, Editor reviewed.

## CRediT authorship contribution statement

**Abdalla Mustafa:** Conceptualization, Methodology, Software, Formal analysis, Writing - original draft. **Nayer N.H. Rizkallah:** Conceptualization, Methodology, Software, Formal analysis, Writing - original draft. **Nehemiah Samuel:** Writing - review & editing. **Shlokarth Balupuri:** Supervision, Writing - review & editing.
